# Betulinic Acid Alleviates Spleen Oxidative Damage Induced by Acute Intraperitoneal Exposure to T-2 Toxin by Activating Nrf2 and Inhibiting MAPK Signaling Pathways

**DOI:** 10.3390/antiox10020158

**Published:** 2021-01-22

**Authors:** Li Kong, Lijuan Zhu, Xianglian Yi, You Huang, Haoqiang Zhao, Yazhi Chen, Zhihang Yuan, Lixin Wen, Jing Wu, Jine Yi

**Affiliations:** 1 Hunan Engineering Research Center of Livestock and Poultry Health Care, Colleges of Veterinary Medicine, Hunan Agricultural University, Changsha 410128, China; kongli@stu.hunau.edu.cn (L.K.); zhulijuan@stu.hunau.edu.cn (L.Z.); yixianglianhj@hotmail.com (X.Y.); huangyou@stu.hunau.edu.cn (Y.H.); zhaohaoqiang2020@stu.hunau.edu.cn (H.Z.); Chenyazhi@stu.hunau.edu.cn (Y.C.); zhyuan2016@hunau.edu.cn (Z.Y.); wenlixinedu@hotmail.com (L.W.); 2 Hunan Co-Innovation Center of Animal Production Safety, Changsha 410128, China

**Keywords:** betulinic acid, T-2 toxin, oxidative stress, MAPK signaling pathway, Nrf2 signaling pathway

## Abstract

T-2 toxin, which is mainly produced by specific strains of *Fusarium* in nature, can induce immunotoxicity and oxidative stress, resulting in immune organ dysfunction and apoptosis. Betulinic acid (BA), a pentacyclic triterpenoids from nature plants, has been demonstrated to possess immunomodulating and antioxidative bioactivities. The purpose of the study was to explore the effect of BA on T-2 toxin-challenged spleen oxidative damage and further elucidate the underlying mechanism. We found that BA not only ameliorated the contents of serum total cholesterol (TC) and triglyceride (TG) but also restored the number of lymphocytes in T-2 toxin-induced mice. BA dose-dependently reduced the accumulation of reactive oxygen species (ROS), enhanced superoxide dismutase (SOD) activity, and decreased malondialdehyde (MDA) content, as well as increased the total antioxidant capacity (T-AOC) in the spleen of T-2-toxin-exposed mice. Moreover, BA reduced inflammatory cell infiltration in the spleen, improved the morphology of mitochondria and enriched the number of organelles in splenocytes, and dramatically attenuated T-2 toxin-triggered splenocyte apoptosis. Furthermore, administration of BA alleviated the protein phosphorylation of p38, c-Jun N-terminal kinase (JNK), and extracellular signal-regulated kinases (ERK); decreased the protein expression of kelch-like erythroid cell-derived protein with CNC homology [ECH]-associated protein1 (Keap1); and increased the protein expression of nuclear factor erythroid 2 [NF-E2]-related factor (Nrf2) and heme oxygenase-1 (HO-1) in the spleen. These findings demonstrate that BA defends against spleen oxidative damage associated with T-2 toxin injection by decreasing ROS accumulation and activating the Nrf2 signaling pathway, as well as inhibiting the mitogen-activated protein kinase (MAPK) signaling pathway.

## 1. Introduction

Mycotoxins are widely found in nature, easily polluting animal feed and the living environment, and have detrimental impacts on humans, animals, and crops, ultimately causing worldwide economic losses [1]. T-2 toxin, generated by *Fusarium*, is regarded as among the most toxic of A-type trichothecene toxins, which often contaminates crops and processed foods and has a wide range of poisonous effects on different species [2,3,4,5]. Animals are exposed to feed contaminated with T-2 toxin through various ways, which causes a variety of toxic effects, mainly including immunotoxicity and cytotoxicity [6]. The spleen, the biggest peripheral immune organ, contains numerous lymphocytes (LYMs) and macrophages. When the spleen is injured, the body immunity sharply reduces. According to related research, T-2 toxin could reduce LYM proliferation, alter membrane function, impair antibody generation, and alter dendritic cell development [7]. Furthermore, a slice of evidence showed that T-2 toxin evokes lymphocyte nuclear chromatin to concentrate and fragment and leads to the apoptosis of lymphoid organs in mice [8]. The above studies proved that T-2 toxin has strong immunotoxicity. T-2 toxin, a protein synthesis inhibitor, can depress peptidyl transferase because of its high binding affinity to the peptidyl transferase of the 60 s ribosomal subunit, resulting in a ribotoxic stress reaction that upregulates c-Jun N-terminal kinase (JNK)/p38 mitogen-activated protein kinases (p38 MAPK) [6]. In addition, T-2 toxin causes oxidative stress and DNA damage, thus activating the apoptotic signaling pathway [9]. Another study verified that T-2 toxin affects the activity of antioxidant enzymes and leads to oxidative stress [10]. These studies indicated that oxidative stress is a feasible mechanism of cellular DNA damage and apoptosis associated with T-2 toxin, which triggers the caspase signaling pathway [9,11,12]. The MAPK family mainly includes JNK, p38, and extracellular-regulated protein kinases (ERKs), which can regulate many physiological activities of eukaryotic cells, such as proliferation, differentiation, and apoptosis [13]. Evidence suggested that JNK, ERK, and p38 proteins were activated in chlorpyrifos (CPF)-treated human neuroblastoma, causing an increase in reactive oxygen species (ROS), subsequently leading to oxidative stress and apoptosis. However, the inhibitor of the MAPK signaling pathway potently ameliorated CPF-induced neurotoxicity [14]. Therefore, JNK, ERK, and p38 MAPK are associated with oxidative stress. Besides, nuclear factor erythroid-2 [NF-E2]-related factor 2 (Nrf2) could activate the antioxidant response element (ARE), leading to the upregulation of heme oxygenase-1 (HO-1), ultimately eliminating oxidative stress [15].

T-2 toxin triggers oxidative stress that causes the occurrence of related diseases, ultimately having a notable impact on the healthy development of the animal industry. Hence, it is particularly important to alleviate oxidative damage for animal health. Antioxidants are substances that can capture and neutralize ROS, thereby ameliorating the ROS-induced damage of cells. Plant-derived antioxidants can effectively maintain the balance of the redox system in animals due to their low toxicity, low residue, and no pollution, which has caught individuals’ attention and led to extensive research.

**Figure 1:**
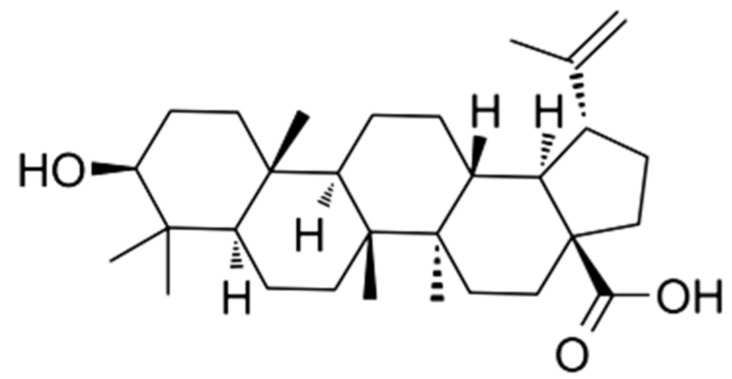
Chemical structure of betulinic acid (BA).

Betulinic acid (BA), a triterpenoid of lupene-structured pentacyclic triterpene (Figure 1), is widely found in plants, such as the birch tree, *Paeonia*, and *Diospyros* [16,17], and has an army of pharmacological activities, including immunomodulation, anti-HIV, antioxidant, antineoplastic, antiobesity, antidyslipidemia, and antianxiety effects. More and more researches have shown that BA has both direct and indirect antioxidation effects by promoting antioxidase activities such as superoxide dismutase (SOD), catalase (CAT), glutathione peroxidase (GSH-Px) activities, decreasing the generation of malondialdehyde (MDA) and ROS in vivo and in vitro [18,19,20]. BA exerts its antioxidative activity by modulating oxidative stress-related pathways. Some experiments demonstrated that BA increased the protein expression of Nrf2/HO-1 and depressed the protein phosphorylation of JNK and p38 in hypoxia/reoxygenation-treated H9c2 cells, indicating that BA exerted its antioxidative activity by regulating Nrf2 and MAPK signaling pathways [21]. BA could also ameliorate the mRNA and protein expression of Nrf2 and HO-1 in experimental membranous nephropathy [22]. Besides, BA could effectively attenuate the oxidative stress of an immune organ induced by dexamethasone (Dex), and the protective effects were associated partly with the regulation of the p38 MAPK pathway [23]. Based on the above research, it is worth exploring whether BA reduces the spleen oxidative impairment challenged by T-2 toxin by mediating Nrf2/MAPK signaling pathways. Hence, the current work aimed to test the protective role of BA and its underlying mechanisms against spleen oxidative damage induced by intraperitoneal injection of T-2 toxin. This may contribute to a viable alternative to alleviate mycotoxin-induced immunotoxicology, thus improving animal health.

## 2. Materials and Methods

### 2.1. Drugs and Reagents

BA (855057) was provided by Sigma (St. Louis, MO, USA). T-2 toxin (MSS1023) was provided by Pribolab (Singapore). The total antioxidant capacity (T-AOC) (A015-1-2), glutathione (GSH) (A006-2-1), MDA (A003-1-2), SOD (A001-3-2), and bicinchonininc acid (BCA) protein commercial kits (A045-4-2) were purchased from Nanjing Jiancheng Bioengineering Institute (Nanjing, Jiangsu, China). Total cholesterol (TC) (BS-300) and triglyceride (TG) (BS-200) kits were provided by Shenzhen Mindray Bio-Medical Electronics (Shenzhen, Guangdong, China). Dihydroethidium (DHE) (GDP1018), the radio immunoprecipitation assay lysis buffer, the terminal deoxynucleotidyl-transferase-mediated dUTP nick end labeling (TUNEL) kit (G1501), hematoxylin-eosin (H&E) staining solution (G1005), an enhanced chemiluminescence (ECL) reagent (G2014), a developing and fixing reagent (G2019), and histone H3 (GB11026) were bought from Wuhan Servicebio (Wuhan, Hubei, China). Diethylether (1009328) was provided by Sinopharm Chemical Reagent (Shanghai, China). Horseradish peroxidase (HRP)-conjugated secondary antibodies (SA00001-2) were provided by Proteintech Inc. (Chicago, IL, USA). The antibodies used in the study, including β-actin (3700S), JNK (9252S), phosphor-JNK (p-JNK) (9251S), p38 (9212S), p-p38 (9211S), ERK (9102S), p-ERK (9101S), kelch-like erythroid cell-derived protein with CNC homology [ECH]-associated protein1 (Keap1) (8047S), Nrf2 (12721S), and HO-1 (82206S), were provided by Cell Signaling Technology Inc. (Danvers, MA, USA). Therm (Fermentas) offered the protein marker (26616) (Burlington, VT, USA).

### 2.2. Animals and Treatment

Fifty male Kunming mice weighing 14–16 g were bought from Hunan Silaike Jingda Laboratory Animal (Changsha, Hunan, China). The animals were kept in a 12 h/12 h light/dark environment with governable humidity (50–70%) and temperature (22–25 °C). The time and dosages of administration (BA and T-2 toxin) were decided according to previous reports [24,25].

Mice were adapted for 1 week prior to the experiment and randomly divided into 5 groups, 10 mice per group, namely control group, T-2 toxin group (4 mg/kg), and BA (0.25, 0.5, or 1 mg/kg) and T-2 toxin co-treatment group. BA was suspended in 1% soluble starch and given by gavage daily for 2 weeks. Meanwhile, control and T-2 toxin groups were administered the same amount of 1% soluble starch. After the last administration of BA, 4 mg/kg of T-2 toxin (T-2 toxin was dissolved in ethanol and then diluted with phosphate buffer solution (PBS)) was injected intraperitoneally to establish a model of oxidative impairment, while animals in the control group were injected intraperitoneally with an equal mixed solution of ethanol and PBS. Fifteen hours after the mice were injected with T-2 toxin, blood samples were collected via venous puncture after light anesthesia of the mice with diethylether. Then the spleen was excised and weighed after the mice were sacrificed and dissected. Subsequently, a part of the spleen was fixed in 4% paraformaldehyde for H&E staining for optical microscope observation or in glutaraldehyde solution for ultrastructural observation by transmission electron microscopy (TEM). Another part of the spleen was frozen in liquid nitrogen for detecting the level of ROS. The rest was stored at −80 °C for antioxidative capacity and Western blot analysis.

### 2.3. Estimation of Blood Biochemical Parameters

Blood samples were put in a refrigerator at 4 °C overnight after being collected from the suborbital vein and then centrifuged at 845× *g* for 10 min. After aspirating the serum, the contents of TC and TG were determined by using the oxidase method and then detected by a Mindray BS-200 automatic biochemistry analyzer (Shenzhen Mindray Bio-Medical Electronics, Shenzhen, Guangdong, China) [20].

### 2.4. Detection of Hematology

An ethylenediaminetetraacetic acid anticoagulant tube was applied to collect blood, and the total number of platelets (PLTs), LYMs, red blood cells (RBCs), and white blood cells (WBCs) was assessed using an automatic blood analyzer (Shenzhen Mindray Bio-Medical Electronics, Shenzhen, Guangdong, China).

### 2.5. Assessment of Intracellular ROS

To evaluate the ROS generation in the spleen, fresh frozen spleen sections were incubated with an oxidative fluorescent dye DHE (5 μM) at 37 °C for 30 min in a light-impermeable moist chamber. The red fluorescence of ethidium converted from DHE was acquired using a fluorescence microscope system (Olympus, Tokyo, Japan) at excitation and emission wavelengths of 535 nm and 610 nm, respectively. The images were captured and quantified by Image-Pro Plus software 6.0 (Media Cybernetics, Silver Spring, MD, USA) [26].

### 2.6. Detection of Antioxidative Capacity

The spleen was homogenized with normal saline into a 10% homogenate and centrifuged at 2500× *g* for 10 min at 4 °C. The supernatant was collected to estimate the antioxidative ability. MDA, a marker of lipid peroxidation, was detected following the thiobarbituric acid method [27]. The GSH level was detected using the dithiodinitrobenzoic acid method, SOD activity was assayed using the xanthine oxidase method, and T-AOC was determined using the colorimetric method, and all indexes were detected following the manufacturer’s protocols [25].

### 2.7. Morphological Examination by H&E Staining

The spleen samples were dehydrated, cleared, embedded in paraffin wax, and sectioned after fixing in 10% neutral-buffered paraformaldehyde. Next, the slices were stained with H&E, and morphological changes in the spleen were observed using an optical microscope (Nikon Eclipse Ci, Tokyo, Japan).

### 2.8. Ultrastructural Observation by TEM

Fresh spleen tissues were fixed in 2.5% glutaraldehyde solution for 3 h and post-fixed in 1% osmium tetroxide for 2 h. The samples were dehydrated, embedded, and sectioned. Uranyl acetate and lead citrate were used to stain the ultrathin sections (60–80 nm). The images were collected and analyzed under TEM (HT7700, Tokyo, Japan).

### 2.9. Observation of Splenocyte Apoptosis by TUNEL Assay

Splenocyte apoptosis was detected by TUNEL assay. The sample slices (4 µm) were permeabilized with proteinase K, rinsed with PBS, and then incubated with TdT and dUTP for 2 h at 37 °C. 4′,6-diamidino-2-phenylindole was used to counterstain the nuclei, and TUNEL-positive cells were stained green. Apoptosis was observed and analyzed under a fluorescence microscope (BA410, Motic, Beijing, China) in five random fields.

### 2.10. Western Blot Analysis

Spleen tissues were processed for Western blot analysis, as described previously [23,25]. After being quantified by a BCA protein assay kit, the samples (30 μg) were run in 12% sodium dodecyl sulfate–polyacrylamide gel electrophoresis and transferred to polyvinylidene difluoride membranes (Millipore, Billerica, MA, USA). The membranes were blocked in 5% nonfat milk solution for 1 h at room temperature and then incubated with the following primary antibodies at 4 °C overnight: p-p38, p38, p-JNK, JNK, p-ERK, ERK, β-actin, Keap1, and HO-1 (1:1000). After being washed three times with tris-buffered saline Tween, the membranes were incubated with HRP-conjugated secondary antibodies (diluted 1:3000) for 1 h. The protein bands were detected with ECL and quantified using AlphaEaseFC software (Alpha Innotech, San Leandro, CA, USA).

The nuclear proteins of histone H3 and Nrf2 in spleen were also detected by Western blot. Briefly, the spleen tissue was homogenized, followed by centrifugation at 211× *g* for 5 min, and then the supernatant that included cytoplasmic protein was discarded. The nuclear protein extraction reagent for mixing with phenylmethanesulfonyl fluoride was added after the residual supernatant was completely absorbed. Cell precipitation was performed by violent rotation on ice at high speed for 15–30 s at intervals of 1–2 min for a total of 30 min. The supernatant was immediately aspirated to obtain nuclear protein after centrifugation at 13,523× *g* for 10 min at 4 °C. The samples were analyzed with Western blot, as described above.

### 2.11. Statistical Analysis

All data were presented as the mean ± standard error of the mean (SEM) and estimated by one-way analysis of variance (ANOVA) and subsequently Duncan’s post hoc test using SPSS 17.0 software (SPSS Inc., Chicago, IL, USA). *p* < 0.05 was regarded as statistically significant.

## 3. Results

### 3.1. Effects of BA on Serum Biochemical Indicators and Blood Indicators in T-2 Toxin-Treated Mice

**Figure 2:**
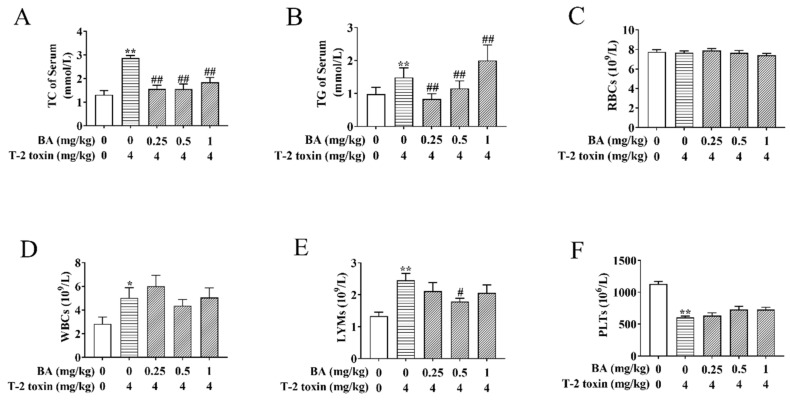
Effects of BA on serum biochemical indicators and blood indicators in T-2 toxin-treated mice. Blood samples were collected, and then the levels of total cholesterol (TC) (**A**) and triglyceride (TG) (**B**) were assayed using the oxidase method. An automatic blood analyzer was used to detect the levels of red blood cells (RBCs) (**C**), white blood cells (WBCs) (**D**), lymphocytes (LYMs) (**E**) and platelets (PLTs) (**F**). Data were presented as the mean ± standard error of the mean (SEM) (10 mice per group). * *p* < 0.05 and ** *p* < 0.01 vs. the control group. ^#^ *p* < 0.05 and ^##^ *p* < 0.01 vs. the T-2 toxin group.

Previously, there was strong evidence that lipoprotein metabolism is related to the immune function and the generation of ROS has to do with abnormal lipid metabolism [28]. To observe the effects of BA on blood lipids in T-2 toxin-treated mice, the contents of TC and TG in serum were determined (Figure 2A,B). BA pretreatment significantly decreased the increase in TC content evoked by T-2 toxin in serum. The 0.25 and 0.5 mg/kg BA pretreatment also remarkedly reduced the level of TG, while 1 mg/kg BA pretreatment increased the content of TG in the serum of T-2 toxin-challenged mice. The above findings suggested that BA has a remission effect on the abnormal serum lipid metabolism triggered by T-2 toxin.

The LYM number in the blood reflects the organism’s immunity, and an increase in the WBC number may be associated with an inflammatory response [29]. Subsequently, the related blood indicators were detected. As per the results shown in Figure 2C–F, there were no obvious differences in the number of RBCs between groups. The numbers of WBCs and LYMs were enhanced, while the number of PLTs reduced in the T-2 toxin-induced group compared to the control group. BA pretreatment markedly decreased the increase in the LYMs number caused by T-2 toxin, while it had no obvious effect on the number of WBCs and platelets. The results implicated that BA can play a certain role in body immunity by regulating the number of LYMs.

### 3.2. BA Protects against Spleen Oxidative Stress in T-2 Toxin-Intoxicated Mice

**Figure 3:**
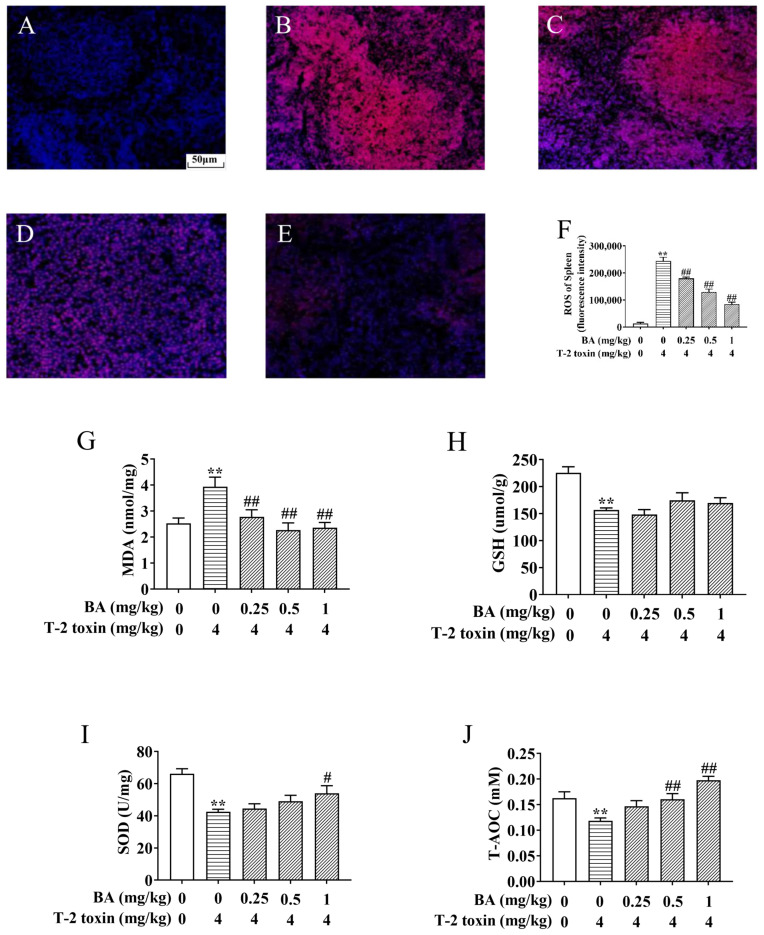
BA protects against T-2 toxin-triggered spleen oxidative stress. The reactive oxygen species (ROS) level in the spleen was determined by a fluorescence microscope using dihydroethidium (DHE). Notes: control group (**A**), T-2 toxin group (**B**), 0.25 mg/kg BA + T-2 toxin group (**C**), 0.5 mg/kg BA + T-2 toxin group (**D**), and 1 mg/kg BA + T-2 toxin group (**E**). Scale bar, 50 μm. Quantitative analysis of ROS level (**F**). The levels of malondialdehyde (MDA) (**G**), glutathione (GSH) (**H**), superoxide dismutase (SOD) (**I**), and total antioxidant capacity (T-AOC) in the spleen, (**J**) Data were presented as the mean ± SEM (10 mice per group). ** *p* < 0.01 vs. the control group. ^#^ *p* < 0.05 and ^##^ *p* < 0.01 vs. the T-2 toxin group.

The spleen, as the body’s largest peripheral immune organ, is one of the main target organs for the action of T-2 toxin [30]. T-2 toxin exposure triggers excessive accumulation of ROS and MDA, leading to oxidative stress. However, the increase in SOD, CAT, and GSH reduces the oxidative damage [9,31]. To explore the protective role of BA against spleen oxidative damage associated with T-2 toxin exposure, the levels of ROS, MDA, SOD, T-AOC, and GSH were estimated. As shown in Figure 3, the generation of ROS and MDA in the spleen of the T-2 toxin group was elevated significantly, but the levels of SOD, GSH, and T-AOC obviously declined, revealing the occurrence of spleen oxidative stress provoked by T-2 toxin. In contrast, BA pretreatment reversed these variations, except the change in GSH wasn’t obvious, which suggested that BA can ameliorate spleen oxidative damage by reducing ROS and lipid peroxidation accumulation and enhancing the antioxidant defense system.

### 3.3. BA Mitigated Spleen Injury in T-2 Toxin-Exposed Mice

**Figure 4:**
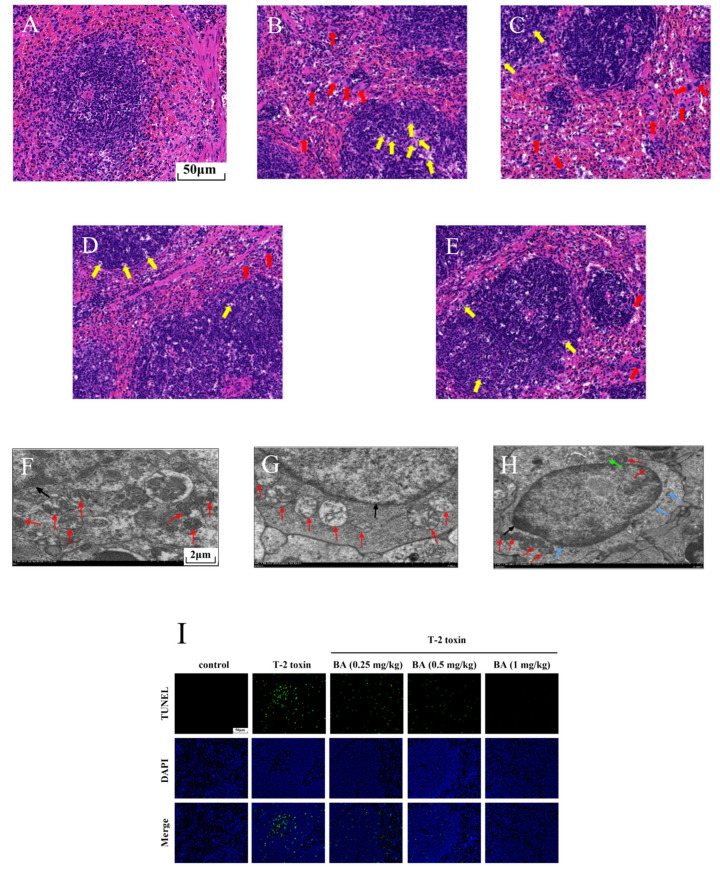

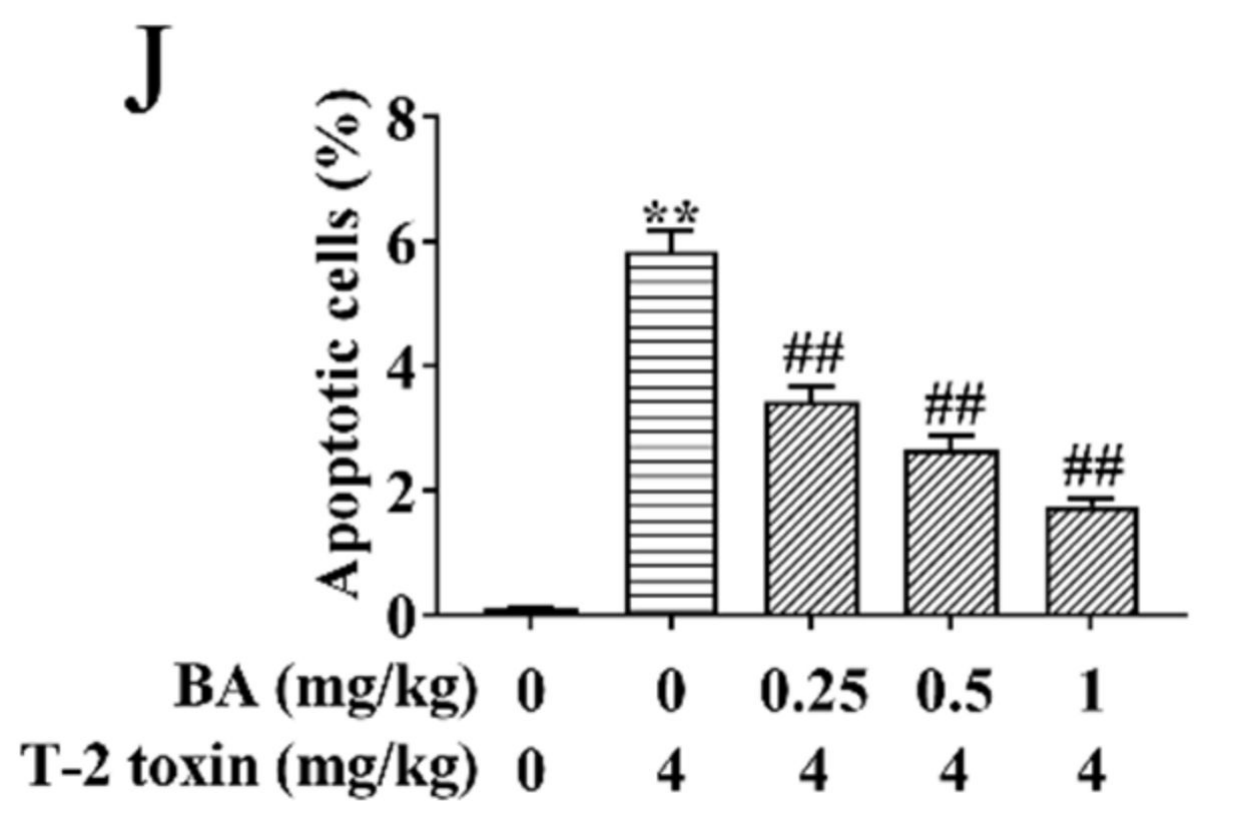
Effects of BA on spleen injury in T-2 toxin-exposed mice. Morphological changes in the spleen were examined using hematoxylin-eosin (H&E) staining. Notes: control group (**A**), T-2 toxin group (**B**), 0.25 mg/kg BA + T-2 toxin group (**C**), 0.5 mg/kg BA + T-2 toxin group (**D**), and 1 mg/kg BA + T-2 toxin group (**E**). Red arrow: macrophages. Yellow arrow: LYMs. Scale bar, 50 μm. The ultrastructure of the spleen was observed using transmission electron microscopy (TEM). Notes: control group (**F**), T-2 toxin group (**G**), and 0.5 mg/kg BA + T-2 toxin group (**H**). Red arrow: mitochondria. Black arrow: nucleus. Green arrow: endoplasmic reticulum. Blue arrow: Golgi apparatus. Scale bar, 2 μm. Splenocyte apoptosis was detected by terminal deoxynucleotidyl-transferase-mediated dUTP nick end labeling (TUNEL) assay (**I**). Scale bar, 50 μm. Percentage of apoptotic cells (**J**). Data were presented as the mean ± SEM (10 mice per group). ** *p* < 0.01 vs. the control group. ^##^ *p* < 0.01 vs. the T-2 toxin group.

To further explore the possible protective effects of BA on spleen injury induced by T-2 toxin, morphological changes were observed using H&E staining (Figure 4A–E). The boundary between the red pulp and the white pulp was clear, the arterial sheath and the spleen trabecula were visible, and the morphological structure was normal in the control group (Figure 4A). While in the T-2 toxin group, a multitude of macrophages were observed in the red pulp, at the same time, there was LYM necrosis in the white pulp because the nucleus fragmented or dissolved (Figure 4B). However, LYM necrosis and macrophage infiltration were ameliorated with BA pretreatment in the spleen (Figure 4C–E).

Mitochondria are not only the organelles for energy production in cells but also the place where cells perform aerobic respiration. In addition to providing energy for cell activities, mitochondria can also participate in various physiological activities, such as cell differentiation and apoptosis [32]. Therefore, we observed the intracellular organelles of the spleen using TEM. As shown in Figure 4F–H, we found that the control group had abundant mitochondria and the ultrastructure was normal. After exposure to T-2 toxin, the mitochondria number decreased significantly, and the mitochondria exhibited swelling and ridge break-up. The organelle number was restored and the morphology of the mitochondria improved after pretreatment with BA (0.5 mg/kg). It was inferred that BA has an improvement effect on the reduced number of splenocyte mitochondria induced by T-2 toxin, which helps to maintain normal physiological activities.

Previous studies have proved that oxidative stress activates the apoptosis signaling pathway and induces apoptosis [32]. Therefore, splenocyte apoptosis was detected using TUNEL assay in this study. Compared to the control group, T-2 toxin dramatically elevated the apoptosis of splenocyte (Figure 4I,J). However, BA pretreatment dramatically reduced T-2 toxin-induced splenocyte apoptosis dose-dependently. The results implied that BA can ameliorate apoptosis by inhibition of oxidative stress.

### 3.4. Effects of BA on the Protein Expression of the MAPK Signaling Pathway in the Spleen of T-2 Toxin-Intoxicated Mice

**Figure 5:**
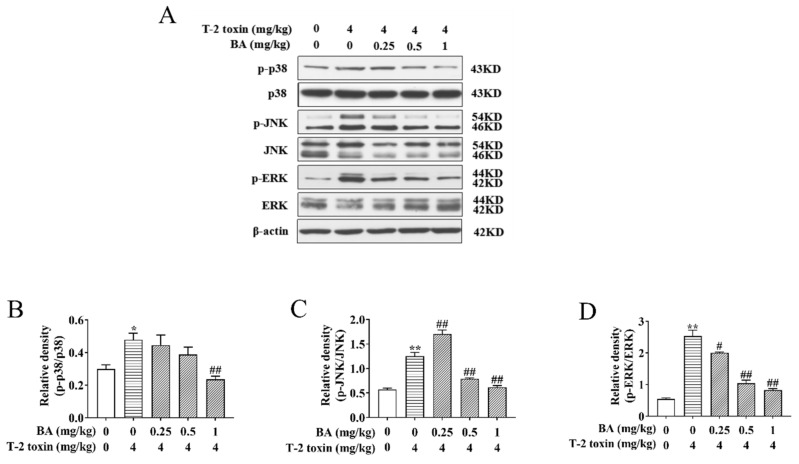
Effects of BA on the relative proteins expression of the mitogen-activated protein kinase (MAPK) signaling pathway in the spleen of T-2 toxin-intoxicated mice. The protein expression and phosphorylation of ERK, JNK, and p38 were evaluated by Western blot (**A**). Protein bands for each region were quantified, and the intensity was normalized to the intensity of the β-actin band present on the same blot. The values were expressed as the ratios of p-p38/p38 (**B**), p-JNK/c-Jun N-terminal kinase (JNK) (**C**), and p-ERK/extracellular signal-regulated kinases (ERK) (**D**). Data were presented as the mean ± SEM (3 mice per group). * *p* < 0.05 and ** *p* < 0.01 vs. the control group. ^#^ *p* < 0.05 and ^##^ *p* < 0.01 vs. the T-2 toxin group.

It has been verified that activation of the MAPK signaling pathway is not only associated with oxidative damage but also plays a key part in the biological signaling transduction of cells [13,14]. To investigate whether BA can reduce oxidative damage by affecting the MAPK signaling pathway, the protein expression and phosphorylation of ERK, JNK, and p38 were analyzed using Western blot. We found that the ratios of p-p38/p38, p-JNK/JNK, and p-ERK/ERK saw a dramatic rise in the T-2 toxin group, while BA treatment lowered the ratios of p-p38/p38 and p-ERK/ERK in a dose-dependent manner. In addition, 0.5 and 1 mg/kg BA pretreatment also reduced the ratio of p-JNK/JNK, but there was marked upregulation of p-JNK/JNK in the BA group at a dosage of 0.25 mg/kg (Figure 5). Results indicated that BA has protective effects against spleen oxidative damage by inhibiting the MAPK signaling pathway.

### 3.5. Effects of BA on the Protein Expression of the Nrf2 Signaling Pathway in the Spleen of T-2 Toxin-Intoxicated Mice

**Figure 6:**
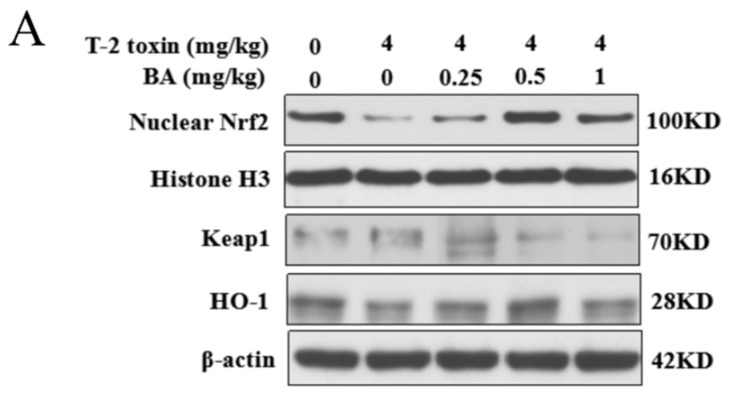

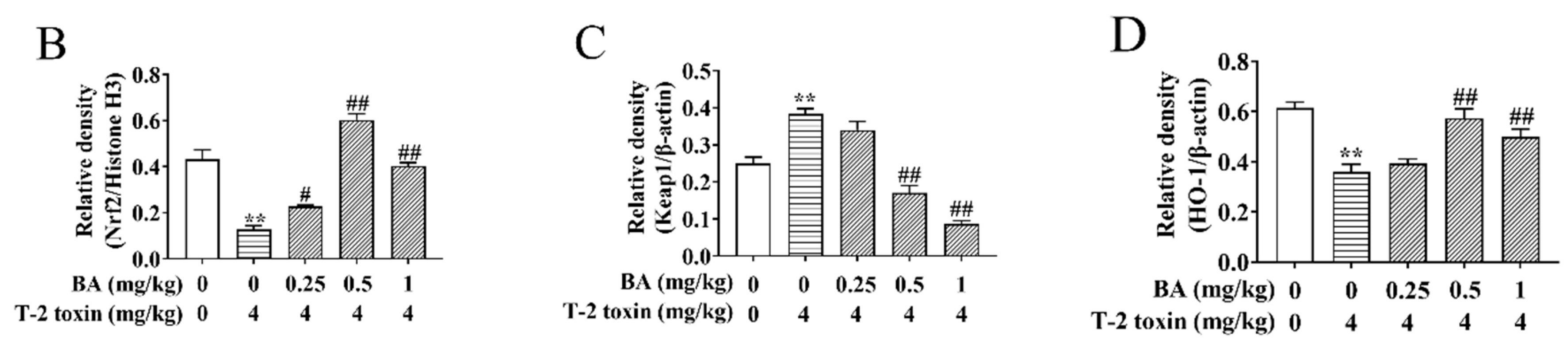
Effects of BA on the relative protein expression of the Nrf2 signaling pathway in the spleen of T-2 toxin-intoxicated mice. The protein expressions of nuclear factor erythroid 2 [NF-E2]-related factor 2 (Nrf2), kelch-like erythroid cell-derived protein with CNC homology [ECH]-associated protein 1 (Keap1), and heme oxygenase-1 (HO-1) were evaluated by Western blot (**A**). Quantitative analysis of Nrf2 (**B**), Keap1 (**C**), and HO-1 (**D**). Data were presented as the mean ± SEM (3 mice per group). ** *p* < 0.01 vs. the control group. ^#^ *p* < 0.05 and ^##^ *p* < 0.01 vs. the T-2 toxin group.

The Nrf2 signaling pathway is a key pathway of the cellular oxidative stress response [21]. As showed in Figure 6, T-2 toxin remarkedly enhanced the protein expression of Keap1 and suppressed the protein expression of Nrf2 and HO-1. Compared to the T-2 toxin group, BA pretreatment, however, remarkedly decreased Keap1 protein expression and accelerated the protein expression of Nrf2 and HO-1 in the spleen of T-2 toxin-stimulated mice. The findings indicated that BA might activate the Nrf2 signaling pathway to protect against spleen oxidative damage triggered by T-2 toxin in mice.

## 4. Discussion

T-2 toxin, *Fusarium*’s secondary metabolite, is liable to contaminate crops, creating a health threat for animals and humans [1]. T-2 toxin has dual functions of immunosuppression and immunostimulation. A study showed that T-2 toxin affects the function of immune organs, immune cells, and immune molecules [33]. It was found that the spleen’s weight in a broiler chicken treated with T-2 toxin decreased, making a dent in its immune system; simultaneously, oxidative injury and cell apoptosis caused by T-2 toxin sharply increased in splenocytes [34]. BA, a naturally occurring pentacyclic triterpenoid, acts as a plant-derived immunomodulator and antioxidant to effectively enhance the antioxidant capacity of immune organs in mice [23,35]. In a study of the protective effect of BA on the testis oxidative damage caused by T-2 toxin, BA exhibited antioxidant activity equivalent to vitamin E [24]. Besides, research reported that the oral administration amount of BA (0.25, 0.5, or 1 mg/kg) posed little injury to mice and even enhanced the immunomodulatory function of the spleen and thymus [35]. BA has a preventive effect against Dex-triggered thymocyte apoptosis, which could be in connection to the antioxidation of BA [32]. Moreover, there is increasing evidence that BA has a pleasurable antioxidative property in a variety of cells [16,17]. In this study, oxidative stress was caused by intraperitoneal injection of T-2 toxin to assess the protective effect of BA and its underlying mechanisms.

A study found that there is interaction between dyslipidemia and immune cells [36]. What’s more, dyslipidemia might result in a variable degree of oxidation [37]. A related study showed that 0.05 mg/kg of T-2 toxin markedly elevates the levels of TC and low-density lipoprotein cholesterol and decreases the level of high-density lipoprotein cholesterol in the liver [38]. Besides, a series of documents have proved that T-2 toxin promotes the process of liver damage, such as hepatocyte apoptosis and necrosis [39,40]. It could be seen from this experiment that T-2 toxin prominently elevated the levels of TC and TG in the serum, which indicated that 4 mg/kg of T-2 toxin causes abnormal serum lipid metabolism in mice. A previous experiment proved that BA has the effect of lowering blood fat by increasing the levels of insulin and leptin to lower the content of TC and TG in plasma [41]. In addition, a study verified that BA can inhibit lipid absorption in the intestine by hindering pancreatic lipase and cholesterol acyltransferase-2 [17]. This finding is consistent with the current study, which discovered that BA administration can significantly reduce the contents of TC and TG. However, 1 mg/kg of BA significantly elevated the TG content, which might be due to individual differences, or 1 mg/kg of BA had no effect on the TG content.

One study reported that T-2 toxin shows hematotoxicity, which reduces PLT and WBC contents in the blood as well as decreases wound coagulation ability and anti-infection ability, causing blood cell apoptosis and bone marrow necrosis [29]. Furthermore, T-2 toxin exhibited immunotoxicity by hurting LYM lines [30]. These results might be associated with the fact that T-2 toxin causes cells’ lipid peroxidation and hinders the synthesis of cellular proteins and DNA [29]. This trial showed that acute exposure to T-2 toxin prominently lowers the PLTs number and elevates the numbers of WBCs and LYMs. WBCs and LYMs belong to immune cells. The increase in WBCs and LYMs contents implied that T-2 toxin gives rise to the occurrence of compensatory immunity and inflammation. Due to the complexity of the immune system, even if the experimental conditions were similar, the immunotoxicity results of T-2 toxin were not completely consistent. Early laboratory research proved that BA regulates LYM proliferation and enhances the phagocytosis of macrophages to strengthen cellular immunity and humoral immunity [42]. The current study found that 0.5 mg/kg of BA pretreatment significantly reduced the LYMs number in blood. However, BA had no dramatic effect on the other blood cells, implying that BA plays an important role in immune regulation by downregulating the number of LYMs.

Oxidative stress has been recognized as one of the main mechanisms in T-2 toxin-provoked toxicity and cell death [11,12]. In rat ovarian granulosa cells, T-2 toxin caused an elevation in ROS and MDA contents and a reduction in SOD, GSH-Px, and CAT levels [43]. A previous study manifested that T-2 toxin administration could decrease antioxidase activity and raise lipid peroxidation in mouse hepatocytes, indicating T-2 toxin has a prominent toxic effect on the liver by induction of oxidative stress [9]. Consistent with the above reports, this study showed that T-2 toxin lowers the contents of GSH, SOD, and T-AOC and enhances the accumulation of MDA and ROS. Our previous studies have shown that BA significantly reduces lipid peroxidation and increases the activities of GSH-Px and CAT in the thymus, which indicates that BA plays a protective role against oxidative damage of lymphoid immune organs caused by Dex [32,42]. Additionally, a previous study affirmed that BA and vitamin E (an antioxidant) restore the intestinal barrier dysfunction triggered by T-2 toxin by regulating the levels of CAT, GSH-Px, GSH, and MDA, as well as inhibiting the inflammation response [44]. Meanwhile, there was a good protection effect in that BA and vitamin E ameliorated T-2 toxin-induced testis oxidative damage [24]. Similar to these studies, pretreatment with BA reversed the above indexes induced by T-2 toxin, suggesting that BA can ameliorate spleen oxidative damage by reducing ROS and lipid peroxidation and enhancing the antioxidant defense system.

Research has proved that T-2 toxin led to oxidative damage in tissues where cells divided actively, mainly including the bone marrow, gastrointestinal tract, spleen, and thymus in chickens [34,45]. Furthermore, a previous study found that BA ameliorated T-2 toxin-provoked testis morphology damage [24]. Similar to the above result, in this experiment, the spleen structure of the T-2 group was disordered, the white pulp was incomplete, and a large number of macrophages and LYMs underwent necrosis, which indicated that T-2 toxin stimulates the inflammatory reaction in the spleen. Meanwhile, the spleen structure of BA pretreatment groups was clear, inflammatory cell infiltration was markedly reduced, and the cells were arranged regularly. This result suggests that BA can reverse the morphological changes in the spleen caused by T-2 toxin to some extent. It is well known that mitochondria are important organelles in eukaryotes, which not only participate in redox homeostasis but also play an important role in apoptosis [46,47]. In addition, some studies have reported that oxidative stress can activate the apoptosis pathway [9,11,12]. The excessive ROS gave rise to mitochondrial oxidative damage and decreased the level of mitochondrial GSH [48]. Mitochondrial dysfunction caused by oxidative damage contributed to the cell apoptosis and necrosis [49]. Previous research showed that T-2 toxin exposure motivated oxidative stress and led to mitochondrial fragmentation, ultimately mediating a mitochondrial-dependent apoptotic pathway in human liver 7702 cells [50]. BA inhibited splenocyte apoptosis induced by Dex by upregulating antioxidant enzymes, decreasing lipid peroxidation, restoring mitochondrial function, and regulating the mitochondrial signaling pathway [51]. Consistent with this study, T-2 toxin significantly decreased the number of mitochondria and remarkedly promoted splenocyte apoptosis in mice. However, BA pretreatment restored the number of organelles in mitochondria. Meanwhile, BA sharply decreased the apoptosis of splenocytes in a dose-dependent manner. The results further illuminated that BA effectively restores T-2 toxin-induced splenocyte apoptosis by enhancing the mitochondrial number and inhibiting oxidative stress.

The MAPK signaling pathway is closely correlated with oxidative stress and plays a crucial part in cell biosignal transmission. The MAPK family includes subfamilies such as JNK, p38, and ERK. Under oxidative stress, excessive ROS activates apoptosis signal-regulating kinase 1 (ASK1) and then activates JNK, p38, and ERK, which are transferred into the nucleus, promoting the transcription and expression of related genes, causing a cascade reaction, which leads to apoptosis [52,53,54]. Previous research has shown that BA reduces LYMs apoptosis by inhibiting the expression of ASK1, JNK, and p38 genes and proteins to protect LYMs from the damage of oxidative stress induced by Dex [32]. In this experiment, pretreatment with BA dramatically downregulated the ratios of p-ERK/ERK, p-p38/p38, and p-JNK/JNK in the spleen of T-2 toxin-exposed mice. It is worth noting that 0.25 mg/kg of BA significantly restrained the protein expression of p-JNK and JNK, yet the ratio of p-JNK/JNK increased. The reason might be that the effect of BA at a 0.25 mg/kg dosage on p-JNK and JNK is not in the most suitable concentration range. However, 0.5 and 1 mg/kg of BA obviously decreased the ratio of p-JNK/JNK in a dose-dependent manner. These results indicated that BA alleviates spleen oxidative damage by inhibiting the phosphorylation expression of JNK, ERK, and p38 in the MAPK signaling pathway.

The Nrf2 signaling pathway regulates the transcription of antioxidation genes and is deemed to be the paramount pathway against oxidative stress [55]. The activation of Nrf2 and the expression of its target gene HO-1 could protect cells from apoptosis, inflammation, and oxidative stress [56]. Therefore, the Nrf2 signaling pathway plays a crucial role in regulating ROS balance and ameliorating oxidative stress. A previous study suggested that BA mitigates myocardial hypoxia/reoxygenation by enhancing the activation of the Nrf2 signaling pathway [21]. In addition, BA inhibited oxidative stress to cure membranous nephropathy by activating Nrf2 expression [22]. In this study, T-2 toxin downregulated the protein expression of Nrf2 and HO-1, while significantly upregulating the protein expression of Keap1. However, BA administration markedly reversed these changes. These results indicated that BA has an antioxidant effect on T-2 toxin-treated mice partly by activating the Nrf2 signaling pathway.

**Figure 7:**
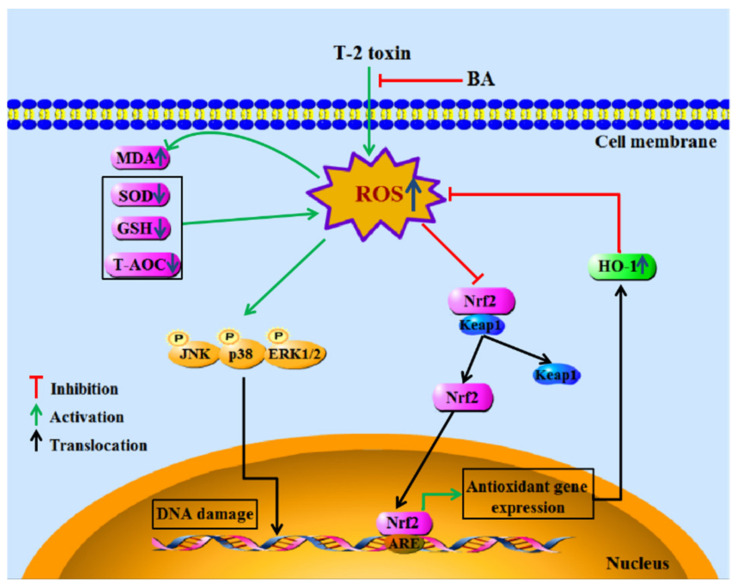
The main mechanism of BA in protecting splenocytes against oxidative damage caused by T-2 toxin.

To sum up, the findings demonstrated the antioxidative and immunomodulatory activities of BA on spleen oxidative damage induced by acute intraperitoneal exposure to T-2 toxin by elevating the activities of antioxidant enzymes, reducing lipid peroxidation and ROS accumulation, and decreasing splenocyte apoptosis via downregulation of the MAPK and upregulation of the Nrf2 signaling pathway (Figure 7). The results show that BA can be potentially used as a detoxicant for T-2 toxin-contaminated feed, thus providing protection to animals against potential immune system damage.

## Data Availability

Not applicable.

## Abbreviations

ARE	antioxidant response element
ANOVA	one-way analysis of variance
ASK1	apoptosis signal-regulating kinase 1
BA	betulinic acid
CPF	chlorpyrifos
CAT	catalase
Dex	dexamethasone
DHE	dihydroethidium
ERK	extracellular signal-regulated kinases
ECL	enhanced chemiluminescence
GSH-Px	glutathione peroxidase
GSH	glutathione
HO-1	heme oxygenase-1
H&E	hematoxylin-eosin
HRP	horseradish peroxidase
JNK	c-Jun N-terminal kinase
Keap1	kelch-like erythroid cell-derived protein with CNC homology [ECH]-associated protein 1
LYMs	lymphocytes
MDA	malondialdehyde
MAPK	mitogen-activated protein kinases
Nrf2	nuclear factor erythroid 2 [NF-E2]-related factor 2
p38	p38 mitogen-activated protein kinase
PLTs	platelets
ROS	reactive oxygen species
RBCs	red blood cells
SOD	superoxide dismutase
SEM	standard error of the mean
TC	total cholesterol
TG	triglyceride
T-AOC	total antioxidant capacity
TUNEL	terminal deoxynucleotidyl-transferase-mediated dUTP nick end labeling
TEM	transmission electron microscopy
WBCs	white blood cells

## References

[1] Zain M.E. (2011). Impact of mycotoxins on humans and animals. J. Saudi. Chem. Soc..

[2] Jaevi V., Wu Q., Nepovimova E., Kua K. (2020). Cardiomyopathy induced by T-2 toxin in rats. Food Chem. Toxicol..

[3] Ling A., Sun L.W., Guo W.B., Sun S.Y., Yang J.H., Zhao Z.H. (2020). Individual and combined cytotoxic effects of T-2 toxin and its four metabolites on porcine Leydig cells. Food Chem. Toxicol..

[4] Yang L.C., Tu D., Wu Y.X., Liu W., Hu Y., Liu T.B., Tan L., Li Y.L., Lei H.Y., Zhan Y. (2020). Distribution and persistence of residual T-2 and HT-2 toxins from moldy feed in broiler chickens. Toxicon.

[5] Yang L., Guo X. (2020). Response to comment on “Comparison of the toxic mechanism of T-2 toxin and deoxynivalenol on human chondrocytes by microarray and bioinformatics analysis”. Toxicol. Lett..

[6] Doi K., Ishigami N., Sehata S. (2008). T-2 toxin-induced toxicity in pregnant mice and rats. Int. J. Mol. Sci..

[7] Hymery N., Sibiril Y., Parent-Massin D. (2006). In vitro effects of trichothecenes on human dendritic cells. Toxicol. In Vitro.

[8] Shinozuka J., Li G., Kiatipattanasakul W., Uetsuka K., Nakayama H., Doi K. (1997). T-2 toxin-induced apoptosis in lymphoid organs of mice. Exp. Toxicol. Pathol..

[9] Chaudhari M., Jayaraj R., Bhaskar A.S.B., Lakshmana R.P.V. (2009). Oxidative stress induction by T-2 toxin causes DNA damage and triggers apoptosis via caspase pathway in human cervical cancer cells. Toxicology.

[10] Chaudhari M., Jayaraj R., Santhosh S.R., Rao P.V.L. (2009). Oxidative damage and gene expression profile of antioxidant enzymes after T-2 toxin exposure in mice. J. Biochem. Mol. Toxicol..

[11] Doi K., Uetsuka K. (2011). Mechanisms of mycotoxin-induced neurotoxicity through oxidative stress-associated pathways. Int. J. Mol. Sci..

[12] Wu Q.H., Wang X., Yang W., Nüssler A.K., Xiong L.Y., Kuča K., Dohnal V., Zhang X.J., Yuan Z.H. (2014). Oxidative stress-mediated cytotoxicity and metabolism of T-2 toxin and deoxynivalenol in animals and humans: An update. Arch. Toxicol..

[13] Johnson G.L., Vaillancourt R.R. (1994). Sequential protein kinase reactions controlling cell growth and differentiation. Curr. Opin. Cell Biol..

[14] Ki Y.W., Park J.H., Lee J.E., Shin I.C., Koh H.C. (2013). JNK and p38 MAPK regulate oxidative stress and the inflammatory response in chlorpyrifos-induced apoptosis. Toxicol. Lett..

[15] Gao Y., Fu R.R., Wang J., Yang X., Wen L.L., Feng J. (2018). Resveratrol mitigates the oxidative stress mediated by hypoxic-ischemic brain injury in neonatal rats via Nrf2/HO-1 pathway. Pharm. Biol..

[16] Ríos J.L., Máñez S. (2018). New pharmacological opportunities for betulinic acid. Planta Med..

[17] Amiri S., Dastghaib S., Ahmadi M., Mehrbod P., Khadem F., Behrouj H., Aghanoori M.R., Machaj F., Ghamsari M., Rosik J. (2020). Betulin and its derivatives as novel compounds with different pharmacological effects. Biotechnol. Adv..

[18] Zheng Z.W., Song S.Z., Wu Y.L., Lian L.H., Wan Y., Nan J.X. (2011). Betulinic acid prevention of d-galactosamine/lipopolysaccharide liver toxicity is triggered by activation of Bcl-2 and antioxidant mechanisms. J. Pharm. Pharmacol..

[19] Peng J., Lv Y.C., He P.P., Tang Y.Y., Xie W., Liu X.Y., Li Y., Lan G., Zhang M., Zhang C. (2015). Betulinic acid downregulates expression of oxidative stress-induced lipoprotein lipase via the PKC/ERK/c-Fos pathway in RAW264.7 macrophages. Biochimie.

[20] Yi J.E., Xia W., Wu J.P., Yuan L.Y., Wu J., Tu D., Fang J., Tan Z.L. (2014). Betulinic acid prevents alcohol-induced liver damage by improving the antioxidant system in mice. J. Vet. Sci..

[21] Wang D., Chen T.Y., Liu F.J. (2018). Betulinic acid alleviates myocardial hypoxia/reoxygenation injury via inducing Nrf2/HO-1 and inhibiting p38 and JNK pathways. Eur. J. Pharmacol..

[22] Sutariya B., Taneja N., Saraf M. (2017). Betulinic acid, isolated from the leaves of *Syzygium cumini* (L.) Skeels, ameliorates the proteinuria in experimental membranous nephropathy through regulating Nrf2/NF-κB pathways. Chem. Biol. Interact..

[23] Zhu L.J., Yi X.L., Zhao J., Yuan Z.H., Wen L.X., Pozniak B., Obminska-Mrukowicz B., Tian Y.N., Tan Z.L., Wu J. (2018). Betulinic acid attenuates dexamethasone-induced oxidative damage through the JNK-P38 MAPK signaling pathway in mice. Biomed. Pharmacother..

[24] Wu J., Yang C.L., Liu J., Chen J.X., Huang C., Wang J., Liang Z., Wen L.X., Yi J.E., Yuan Z.H. (2019). Betulinic acid attenuates T-2-toxin-induced testis oxidative damage through regulation of the JAK2/STAT3 signaling pathway in mice. Biomolecules.

[25] Zhu L.J., Yi X.L., Ma C.Y., Luo C.X., Kong L., Lin X., Gao X.Y., Yuan Z.H., Wen L.X., Li R.F. (2020). Betulinic acid attenuates oxidative stress in the thymus induced by acute exposure to T-2 toxin via regulation of the MAPK/Nrf2 signaling pathway. Toxins.

[26] Zhu S.Y., Wang Y.X., Wang X.Y., Li J.Y., Hu F. (2014). Emodin inhibits ATP-induced IL-1β secretion, ROS production and phagocytosis attenuation in rat peritoneal macrophages via antagonizing P2X7 receptor. Pharm. Biol..

[27] Woo Y., Lim J.S., Oh J., Lee J.S., Kim J.S. (2020). Neuroprotective effects of euonymus alatus extract on scopolamine-induced memory deficits in mice. Antioxidants.

[28] Delgado R.L., Acosta M.E., Fraga P.A., Bécquer V.M.A., Soto L.Y., Falcón C.V., Vázquez L.A.M., Martínez-Sánchez G., Fernández-Sánchez E. (2012). Lipofundin-induced hyperlipidemia promotes oxidative stress and atherosclerotic lesions in new zealand white rabbits. Int. J. Vasc. Med..

[29] Parent-Massin D. (2004). Haematotoxicity of trichothecenes. Toxicol. Lett..

[30] Minervini F., Fornelli F., Lucivero G., Romano C., Visconti A. (2005). T-2 toxin immunotoxicity on human B and T lymphoid cell lines. Toxicology.

[31] Ahmed A.S.A., Eryilmaz R., Demir H., Aykan S., Demir C. (2019). Determination of oxidative stress levels and some antioxidant enzyme activities in prostate cancer. Aging Male.

[32] Yi J.E., Zhu R.C., Wu J.P., Wu J., Xia W., Zhu L.J., Jiang W.W., Xiang S.T., Tan Z.L. (2016). In vivo protective effect of betulinic acid on dexamethasone induced thymocyte apoptosis by reducing oxidative stress. Pharmacol. Rep..

[33] Krishnamoorthy P., Vairamuthu S., Balachandran C., Muralimanohar B. (2007). Pathology of lymphoid organs in chlorpyriphos and T-2 toxin fed broiler chicken. Int. J. Poult. Sci..

[34] Chen Y.Q., Han S.S., Wang Y., Li D.Y., Zhao X.L., Zhu Q., Yin H.D. (2019). Oxidative stress and apoptotic changes in broiler chicken splenocytes exposed to T-2 toxin. Biomed. Res. Int..

[35] Yi J.E., Obminska-Mrukowicz B., Yuan L.Y., Yuan H. (2010). Immunomodulatory effects of betulinic acid from the bark of white birch on mice. J. Vet. Sci..

[36] Lacy M., Atzler D., Liu R.Q., De W.M., Weber C., Lutgens E. (2019). Interactions between dyslipidemia and the immune system and their relevance as putative therapeutic targets in atherosclerosis. Pharmacol. Ther..

[37] Acharya P., Talahalli R.R. (2019). Aging and hyperglycemia intensify dyslipidemia-induced oxidative stress and inflammation in rats: Assessment of restorative potentials of ALA and EPA + DHA. Inflammation.

[38] Kang R., Perveen A., Li C. (2020). Effects of maternal T-2 toxin exposure on the hepatic glycolipid metabolism in young mice. Ecotoxicol. Environ. Saf..

[39] Wu J., Zhou Y., Yuan Z., Yi J., Chen J., Wang N., Tian Y. (2019). Autophagy and apoptosis interact to modulate T-2 toxin-induced toxicity in liver cells. Toxins.

[40] Liu A., Sun Y., Wang X., Ihsan A., Tao Y., Chen D., Peng D., Wu Q., Wang X., Yuan Z. (2019). DNA methylation is involved in pro-inflammatory cytokines expression in T-2 toxin-induced liver injury. Food Chem. Toxicol..

[41] De M.C.L., Queiroz M.G.R., Arruda F.A.C.V., Rodrigues A.M., De S.D.F., Almeida J.G.L., Pessoa O.D.L., Silveira E.R., Menezes D.B., Melo T.S. (2009). Betulinic acid, a natural pentacyclic triterpenoid, prevents abdominal fat accumulation in mice fed a high-fat diet. J. Agric. Food Chem..

[42] Yi J.E., Lis M., Szczypka M., Obmińska-Mrukowicz B. (2012). Influence of betulinic acid on lymphocyte subsets and humoral immune response in mice. Pol. J. Vet. Sci..

[43] Wu J., Tu D., Yuan L.Y., Yuan H., Wen L.X. (2013). T-2 toxin exposure induces apoptosis in rat ovarian granulosa cells through oxidative stress. Environ. Toxicol. Pharmacol..

[44] Luo C.X., Huang C.L., Zhu L.J., Kong L., Yuan Z.H., Wen L.X., Li R.F., Wu J., Yi J.E. (2020). Betulinic acid ameliorates the T-2 toxin-triggered intestinal impairment in mice by inhibiting inflammation and mucosal Barrier dysfunction through the NF-κB signaling pathway. Toxins.

[45] Jaradat Z.W., Viià B., Marquardt R.R. (2006). Adverse effects of T-2 toxin on chicken lymphocytes blastogenesis and its protection with Vitamin E. Toxicology.

[46] Circu M.L., Aw T.Y. (2010). Reactive oxygen species, cellular redox systems, and apoptosis. Free Radic. Biol. Med..

[47] Tait S.W.G., Green D.R. (2013). Mitochondrial regulation of cell death. CSH. Perspect. Biol..

[48] Xiao J., Zhang R.F., Huang F., Liu L., Deng Y.Y., Ma Y.X., Wei Z.C., Tang X.J., Zhang Y., Zhang M.W. (2017). Lychee (*Litchi chinensis Sonn.*) pulp phenolic extract confers a protective activity against alcoholic liver disease in mice by alleviating mitochondrial dysfunction. J. Agric. Food Chem..

[49] Zhang G.W., Yang W., Jiang F., Zou P., Zeng Y.F., Ling X., Zhou Z.Y., Cao J., Ao L. (2019). PERK regulates Nrf2/ARE antioxidant pathway against dibutyl phthalate-induced mitochondrial damage and apoptosis dependent of reactive oxygen species in mouse spermatocyte-derived cells. Toxicol. Lett..

[50] Yang J.H., Guo W.B., Wang J.H., Yang X.L., Zhang Z.Q., Zhao Z.H. (2020). T-2 toxin-induced oxidative stress leads to imbalance of mitochondrial fission and fusion to activate cellular apoptosis in the human liver 7702 cell line. Toxins.

[51] Yi J., Zhu R., Wu J., Wu J., Tan Z. (2015). Ameliorative effect of betulinic acid on oxidative damage and apoptosis in the splenocytes of dexamethasone treated mice. Int. Immunopharmacol..

[52] Liang T., Zhang X.J., Xue W.H., Zhao S.F., Zhang X., Pei J.Y. (2014). Curcumin induced human gastric cancer BGC-823 cells apoptosis by ROS-mediated ASK1-MKK4-JNK stress signaling pathway. Int. J. Mol. Sci..

[53] Soga M., Matsuzawa A., Ichijo H. (2012). Oxidative stress-induced diseases via the ASK1 signaling pathway. Int. J. Cell Biol..

[54] Szuster-Ciesielska A., Plewka K., Daniluk J., Kandefer-Szersze M. (2011). Betulin and betulinic acid attenuate ethanol-induced liver stellate cell activation by inhibiting reactive oxygen species (ROS), cytokine (TNF-α, TGF-β) production and by influencing intracellular signaling. Toxicology.

[55] Cui G.Z., Luk S.C.W., Li R.A., Chan K.K.K., Lei S.W., Wang L., Shen H.F., Leung G.P.H., Lee S.M.Y. (2015). Cytoprotection of baicalein against oxidative stress-induced cardiomyocytes injury through the Nrf2/Keap1 pathway. J. Cardiovasc. Pharm..

[56] Ren Y.S., Zheng Y., Duan H., Lei L., Deng X., Liu X.Q., Mei Z.N., Deng X.K. (2020). Dandelion polyphenols protect against acetaminophen-induced hepatotoxicity in mice via activation of the Nrf2/HO-1 pathway and inhibition of the JNK signaling pathway. Chin. J. Nat. Med..

